# The influence of glycated hemoglobin in a study comparing patients who required hospitalization or not

**DOI:** 10.1186/1758-5996-7-S1-A95

**Published:** 2015-11-11

**Authors:** Beatriz Dal Santo Francisco Bonamichi, João Eduardo Nunes Salles, Rubens Aldo Sargaço, Adriano Namo Cury

**Affiliations:** 1 Irmandade Santa Casa de Misericórdia de São Paulo, São Paulo, Brazil

## Background

Hyperglycemia can cause deleterious effects in the body as inflammation. The glycated hemoglobin (HbA1c) plays a key role in monitoring of glycemic for reporting the retrospective index of plasma glucose.

## Objective

To evaluate the HbA1c as a prognostic marker in relation to need or not of hospitalization in patients with other diseases.

## Method

A study conducted at the Hospital Samaritano lasting five yrs. The HbA1c was analyzed outpatient and hospitalized for various clinical conditions. With regard to inpatients, according to capillary hyperglycemia observed at admission, verified by a Glucose approved by the FDA (Food and Drug Administration) Precision XTra (Abbott) was collected HbA1c, according to NUMAD Protocol (Multidisciplinary Center for Assistance the diabetic). It was considered hyperglycemia blood glucose values> 140 mg dl-, according to the American Diabetes Association. Outpatients collected according to routine medical request. HbA1c was analyzed by High Performance Liquide Chromatography (HPLC) method. Parameters were established for analysis of glycated hemoglobin equal to or less than 6.9%, 7 to 9%, and greater than 9%. Statistical differences were considered significant at p <0.05.

## Result

Evaluated HbA1c of 2433 inpatients and outpatients 48,164. It was also found adherence to hospital protocol, starting with 300 requests, progressing to the last year with 656 requests HbA1c for patients with hospital hyperglycemia. The average HbA1c in inpatients and outpatients were respectively less than or equal to 6.9%: 57% and 90.58%, 7-9%: 25.55% and 7.72%, greater than 9%: 17.02% and 1.70%.

## Conclusion

The Diabetes Control and Complications Trial (DCCT) and United Kingdom Prospective Diabetes Study (UKPDS), determined the use of HbA1c as a laboratory parameter in control of DM. It is known that the HbA1c can be used as a parameter in determining the risk of progression to microvascular and macrovascular disease in DM, however, little is known about the involvement of other diseases and their complications. A well established hospital protocol can be a critical tool in controlling blood glucose by checking HbA1c. Our study demonstrated that patients with higher HbA1c have greater need for hospitalization besides the pathology, evidencing this method as an important prognostic marker and predictive of need for hospitalization.

